# Dynamic Model for Life History of Scyphozoa

**DOI:** 10.1371/journal.pone.0130669

**Published:** 2015-06-26

**Authors:** Congbo Xie, Meng Fan, Xin Wang, Ming Chen

**Affiliations:** 1 School of Mathematics and Statistics, Northeast Normal University, Changchun, Jilin, P. R. China; 2 College of Science, Dalian Nationalities University, Dalian, Liaoning, P. R. China; Shanxi University, CHINA

## Abstract

A two-state life history model governed by ODEs is formulated to elucidate the population dynamics of jellyfish and to illuminate the triggering mechanism of its blooms. The polyp-medusa model admits trichotomous global dynamic scenarios: extinction, polyps survival only, and both survival. The population dynamics sensitively depend on several biotic and abiotic limiting factors such as substrate, temperature, and predation. The combination of temperature increase, substrate expansion, and predator diminishment acts synergistically to create a habitat that is more favorable for jellyfishes. Reducing artificial marine constructions, aiding predator populations, and directly controlling the jellyfish population would help to manage the jellyfish blooms. The theoretical analyses and numerical experiments yield several insights into the nature underlying the model and shed some new light on the general control strategy for jellyfish.

## Introduction

Jellyfish are among the most conspicuous animals widely distributing in the oceans. They have complex life histories and demographics and plays an essential role in the marine ecosystems acting as a ‘keystone’ species [1]. Jellyfish has been abnormally blooming and flourishing in many waters since 1980s [2–8]. Bloom or large swarm is usually used for a large group of jellyfish that gather in a small area, but may also have a time component, referring to seasonal increases, or numbers beyond what was expected. Blooms are distinct-varying in species composition and size. For example, in Japan, blooms should be > 2,000 medusae of *Nemopilema nomurai* entrapped per set net per day [9], while in the Mediterranean, blooms should be up to 600 medusae of *Pelagia noctiluca* per m^3^ [7]. Jellyfish blooms cause extreme problems to both marine ecosystems and human enterprises [3, 8, 10, 11].

**Fig 1:**
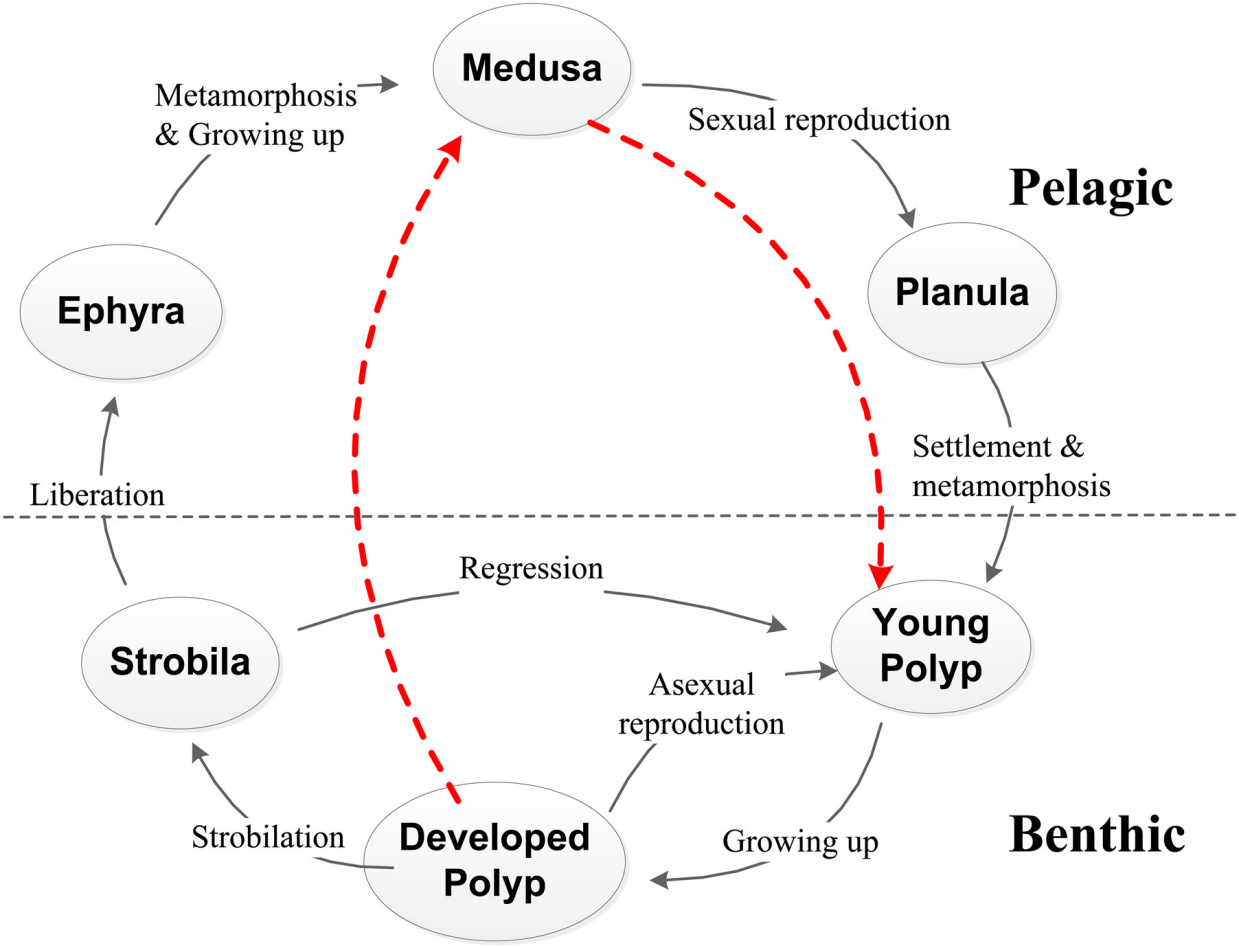
Schematic diagrams of general life cycle of a scyphozoan jellyfish. The life history usually comprises two stages (pelagic and benthic) with several different phases (planula, polyp, strobila, ephyra, and medusa). The solid lines with arrows depict the natural life cycle. The dash line from Medusa to Young Polyp represents the supplement involving sexual reproduction by medusa, settlement and metamorphosis by planula. The dash line from Developed Polyp to Medusa indicates the progress of strobilation, liberation and growing up.

The most metagenetic scyphozoan has complicated life history (Fig 1). Extensive studies [12–16] demonstrate that the early developmental stages in the life cycle play a critical role in the development of jellyfish outbursts. Research is crucial on expounding jellyfish ecology and characterizing its complex life history.

The causes of jellyfish blooms are still uncertain but heavily related to the anthropogenic impact [10, 17] such as overfishing [3, 6, 11], eutrophication [18], climate change [19], habitat modification [20, 21] and alien invasion [22–24]. Those factors directly or indirectly irritate the modes of reproduction of medusae and polyps. For example, overfishing results in the decrease of predators and competitors of medusae [1]; eutrophication brings about abnormal phytoplankton blooms, ocean acidification and hypoxia, which damage the fish but have no effect on jellyfish [4, 25–27]; climate warming accelerates the budding and triggers the strobilation, and, in particular, the temperature usually has determinant effect on the variation and expanding of jellyfish population in the early life stages [16, 28, 29], even “the warmer the better” [30]; habitat modification, i.e. global ocean sprawl of artificial substrates [31], provides suitable habitat for the benthic stage of the life history and has been potentially linked to huge polyp populations and medusa blooms [20, 21]; the alien jellyfish is usually invasive and increasing dramatically, exerts tremendous pressure on native species and shifts the pelagic ecosystem structure seriously [7, 23, 24]. Jellyfish populations definitely respond to those changes from human’s activities. Yet the general awareness of these phenomena is still embryonic and few (theoretical) studies are available [7].

In the course of 20 century, the studies of aquatic system have changed significantly and shifted from purely descriptive disciplines to sciences that champion quantitative approaches and aim to reveal mechanistic relationships [32]. In particular, the use of mathematical formulations and models has emerged as a means to better describe, understand and predict the dynamical properties in aquatic systems. In the paper, we will propose a two-dimensional dynamic model governed by ordinary differential equations to characterize the dynamics of the life history of scyphozoa and provide insight into the mechanisms of jellyfish blooms.

Among the existing models for jellyfish, most are of a statistical nature investigating correlations between environmental indices and abundance [33, 34] or statistical analyses for the changes in jellyfish abundance over time [35, 36]. Others are food chain or food net models governed by system of differential equations based on the energy flowing [37–39]. However, except that [40] modeled the dynamics of a polyp population in a laboratory experiment by the logistic model, there is less dynamic modeling approach to identify the mechanisms forcing the population dynamics of jellyfish through their impact on the life cycle.

The aim of this paper is threefold. First, we expound the long term dynamics of jellyfish by formulating a two-dimensional dynamic model illustrating the scyphozoan life history. Second, we investigate the significant effect of some biotic and abiotic factors such temperature, substrate, and predation on the population dynamics and characterize their key sensitive impacts. Third, we discuss the mechanisms leading to the rapid expansion of jellyfish population even when there is only a few invaders.

## Methods

In this section, a dynamic model is formulated to quantitatively characterize the dynamics of the life history of scyphozoa and to expound the mechanisms driving the variation of population with changing environment.

The life history of most scyphozoan jellyfish comprises several phases (Fig 1). Medusae are dioecious, with the sperm and eggs which combine to produce a planula. The planulae typically settle to the bottom and then metamorphoses into tentacle-bearing polyps (or scyphistomae). The sessile scyphistoma reproduce asexually by budding, stolon, and formation of podocysts. The segmented parts of the strobilating polyp (strobila) develop into ephyra (incipient medusae) that eventually break loose and become ephyrae. After ephyrae are released, strobilae regress to initial scyphistomae and the ephyra grows rapidly into an adult medusa, completing the life cycle.

In this study, our main purpose is to elucidate the population dynamics and the impacts of some environmental factors. We solely focus on the abundance but not on the somatic growth or morphological change of scyphozoan. The planula is the larval stage of polyp and the ephyra is incipient medusa. Either planula and polyp or ephyra and medusa are coincident in the quantity but are different only in the survival rate. That is to say, the number of polyps recruited by planulae is equal to the number of surviving planulae, similarly numbers of medusa are linearly correlated with that of supplementary ephyrae. Moreover, because the number from strobila to young polyp by the regression does not change and the difference between young polyp and developed polyp is only in shape, all phases in the benthic stage including young polyp, developed polyp, and strobila can be integrated into polyp and the change in the number of the benthic polyps only comes from asexual reproduction. In addition, polyp and medusa are two main stages of the life cycle of jellyfish [13], so we assume that the life cycle is simplified to an alternation between the bottom-dwelling polyp (benthic stage) and free-swimming medusa (pelagic stage). Moreover, the asexual reproduction rate and strobilation rate are assumed to be functions of temperature and the resource is sufficient for the jellyfish growth.

Let *P*(*t*) and *M*(*t*) be the population size or number of polyps and medusae at time *t*, respectively. The abundance of polyps changes due to asexual reproduction (*α*(*T*)*P*), recruitment from planulae by sexual reproduction (*s*_1_ *γM*), natural death (*d*_1_ *P*), mortality being covered by silt or consumed by nudibranch (*d*_2_ *P*), and intraspecific competition for substrates (*b*_1_ *P* ^2^). The change of medusae allows for the recruitment of ephyrae via strobilation (*s*_2_ *β*(*T*)*nP*), natural death (*d*_3_ *M*), mortality due to predation from other species (*d*_4_ *M*), and intraspecific competition (*b*_2_ *M* ^2^).

Based on the above framework, the two-state model for scyphozoan growth, different from the classical Kolmogorov model [41], is given by

{dPdt=α(T)P+s1γM-d1P-d2P-b1P2,dMdt=s2β(T)nP-d3M-d4M-b2M2.} (1)

Parameters for the governing system (1) are listed in Table 1 with biological description, ranges from references, and default values used for numerical simulations. The parameter ranges are estimated or evaluated from the empirical and field experiments in references after standardized by days. The details of the standardization are presented in S1 File.

**Table 1:** Parameters of system (1) with ranges and default values used for numerical studies.

Para.	Description	Ranges	Ref.	Unit	V1	V2
*α*(*T*)	Asexual reproduction rate affected by temperature, including budding, stolon and podocyst et al.	0.03 ∼ 0.15^*a*^	[42]	ind ⋅ d^−1^ *P* ^−1^	0.12	0.15
*β*(*T*)	Strobilation rate affected by temperature	0.065 ∼ 0.139^*a*^	[42]	ind ⋅ d^−1^time^−1^ *P* ^−1^	0.108	0.122
*γ*	Sexual reproduction rate	19 ∼ 178^*a*^	[43], [44]	ind ⋅ d^−1^ *M* ^−1^	100	170
*s*_1_	Survival and successful settlement rate of planula	0.001 ∼ 0.3^*b*^	[44, 45]	no unit	0.008	0.01
*s*_2_	Survival rate of ephyra	0.01 ∼ 0.8^*b*^	[44]	no unit	0.2	0.8
*n*	Strobilation times	1 ∼ 2	[42]	times	1	1
*d*_1_	Natural mortality rate of polyp	0 ∼ 0.028^*a*,*b*^	[42], [46]	d^−1^	0.0001	0.0001
*d*_2_	Mortality rate of polyp for being covered by silt or consumed by the nudibranch	0.0001 ∼ 0.3^*b*^	[44]	d^−1^	0.01	0.01
*d*_3_	Natural mortality rate of medusa	0.004 ∼ 0.02^*a*^	[44], [47]	d^−1^	0.006	0.004
*d*_4_	Mortality rate of medusa induced by predation	0.0001 ∼ 0.8^*b*^	[1]	d^−1^	0.08	0.0001
*b*_1_	Intraspecific competition between polyps	0.00001 ∼ 0.1^*b*^	[28, 48]	d^−1^ind^−1^	0.0012	0.0001
*b*_2_	Intraspecific competition between medusae	0 ∼ 0.1^*b*^		d^−1^ind^−1^	0.0001	0.0001

Values marked by ^*a*^ are from experimental data with unit conversion and those marked by ^*b*^ are estimated from references. The details are presented in S1 File.

### Analysis

In this section, we analyze the global dynamics of the governing system using standard qualitative analysis techniques. To facilitate the discussion below, let *a* = *α*(*T*) − *d*_1_ − *d*_2_, *b* = *s*_1_ *γ*, *c* = *s*_2_ *β*(*T*)*n*, and *d* = *d*_3_ + *d*_4_. Then the final form of the model we investigate is

{dPdt=aP+bM-b1P2≔F1(P,M),dMdt=cP-dM-b2M2≔F2(P,M).} (2)

where *a* ∈ ℝ, *b* ≥ 0, *c* ≥ 0, *d* > 0, *b*_1_ > 0, *b*_2_ > 0.

Let *l* = (∣*a*∣*d* + *bc*)/*b*_1_ *d*. Then $\Omega ≔\left\{(P,M)\in {\mathbb{R}}_{+}^{2}:\;0<P<l,0<M<cl/d\right\}$ is positively invariant with respect to system (2). The Dulac’s criterion precludes the existence of nontrivial periodic solutions in Ω. System (2) possibly admits several different equilibrium states: extinction *E*_0_(0, 0), partial survival *E*_1_(*a*/*b*_1_, 0), and coexistence E*(P*, M*). The standard qualitative analysis techniques help to characterize the existence and stability of those equilibria and then the global dynamics of system (2) (see Table 2 for summarization and the S2 File for details of the proof).

**Table 2:** Global dynamics of system (2).

Conditions	Stability	Bifurcation
*ad* + *bc* < 0	*E*_0_ is GAS	
	*E*_1_ and E* do not exist	
*ad* + *bc* = 0	*E*_0_ is saddle-node	saddle-node bifurcation
	*E*_1_ and E* do not exist	
*ad* + *bc* > 0, *c* = 0	*E*_0_ is a saddle	a heteroclinic orbit connecting *E*_0_ and *E*_1_ (see Fig 2(a))
	*E*_1_ is GAS	
	E* do not exist	
*ad* + *bc* > 0, *c* ≠ 0	*E*_0_ is a saddle	a heteroclinic orbit connecting *E*_0_ and E* (see Fig 2(b))
	*E*_1_ does not exist	
	E* is GAS	

Here GAS denotes globally asymptotically stable.

The extinction equilibrium *E*_0_ always exists and is globally asymptotically stable if *ad* + *bc* < 0. In fact, *ad* + *bc* < 0 implies *a* < 0, i.e., *α*(*T*) < *d*_1_ + *d*_2_, which implies that the cloning rate of polyp is less than its mortality rate. In addition, *ad* + *bc* < 0 indicates that the loss dominates recruitment and hence the system is extinct. If *ad* + *bc* ≥ 0, then *E*_0_ is unstable and saddle-node bifurcation occurs when *ad* + *bc* = 0.

**Fig 2:**
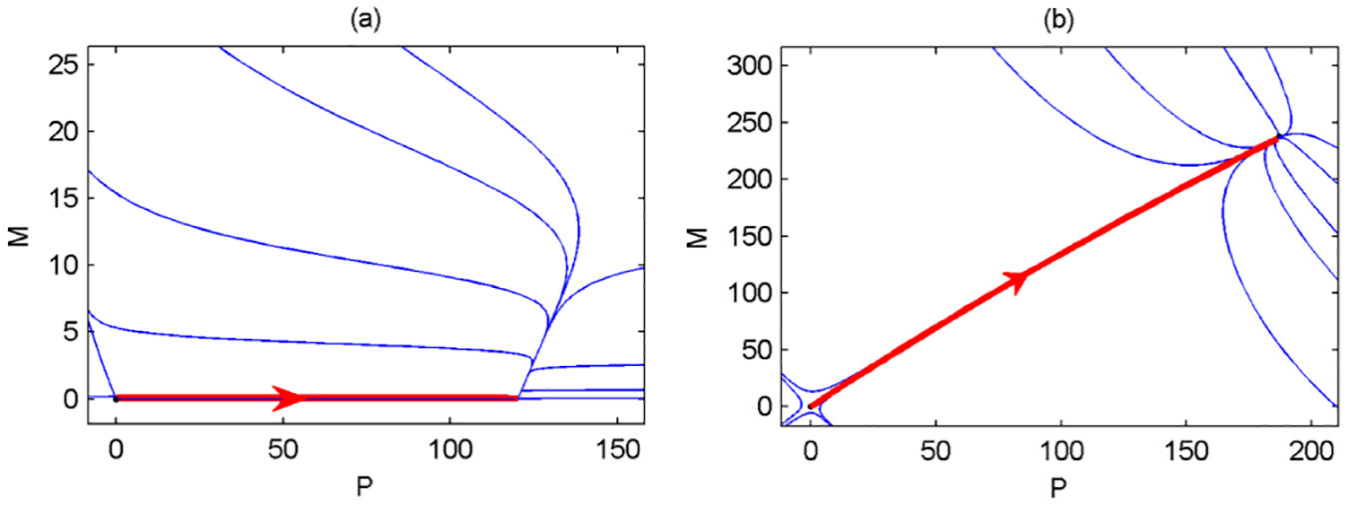
Phase portrait of system (2). (a) *ad* + *bc* > 0, *c* = 0. There are two equilibria with *E*_0_ being unstable and *E*_1_ being globally asymptotically stable. There is a heteroclinic orbit from *E*_0_ to *E*_1_. (b) *ad* + *bc* > 0, *c* ≠ 0. There are two equilibria with *E*_0_ being unstable and E* being globally asymptotically stable and there is a heteroclinic orbit from *E*_0_ to E*.

If *ad* + *bc* > 0 and *c* = 0, i.e., *c* = 0 and *a* > 0, then the boundary equilibrium *E*_1_(*a*/*b*_1_, 0) exists and is globally asymptotically stable. In fact, *c* = *s*_2_ *β*(*T*)*n* = 0 implies that there is no survival of the ephyrae or the temperature is insufficient to strobilate. In such scenario, the polyps would be sleeping in the seabed for a long time and there is no supplement from polyp to medusa. Therefore, the system will stabilize at the boundary equilibrium *E*_1_(*a*/*b*_1_, 0) and there is a heteroclinic orbit connecting *E*_0_ with *E*_1_ (see Fig 2(a)).

The positive equilibrium E*(P*, M*) exists and is globally asymptotically stable if and only if *ad* + *bc* > 0 and *c* ≠ 0. Moreover, there is a heteroclinic orbit connecting *E*_0_ and E* (Fig 2(b) illustrating such a heteroclinic orbit connecting the two equilibrium points for a set of physiologically relevant parameters). E* being globally asymptotically stable implies that both polyp and medusa will eventually survive whatever the initial state of the system is. In fact, M* ∈ (0, *cl*/*d*), P* ∈ (0, *l* + *a*/*b*_1_) if *a* ≤ 0, and P* ∈ [*a*/*b*_1_, *l*) if *a* > 0.

Obviously, with the decreasing of *b*_1_ (intraspecific competition of polyps) or *d* (mortality of medusae), i.e., the increasing of *l*, the upper bound of both P* and M* will sharply increase, and the number of polyps and medusae will burst even when the initial population sizes are at very low levels. This can help to explain why only a few invaders can colonize, multiply, even become the dominant species in some sense. All of these findings are also supported by numerical simulations below.

In particular, if *ad* + *bc* > 0, *c* ≠ 0, and *b* = 0, E*(P*, M*) takes the form of $E^{*}\left(a/b_{1},\left(-b_{1}d+\sqrt{b_{1}^{2}d^{2}+4acb_{1}b_{2}}\right)/2b_{1}b_{2}\right)$. In this case, *b* = *s*_1_γ = 0 indicates that there is no survival of the planulae or no sexual reproduction, in other words, there is no recruitment from the pelagic to the benthic stage. However, the existence of budding et al. can expand the population of polyps in the benthos. The above findings show that the benthic stage is more crucial and more control effort should be implemented in benthic stage.

### Simulation

In order to characterize the effect of temperature, substrate, and predator on the population dynamics of jellyfish, we deliberately investigate a specific jellyfish *Aurelia* sp.. The reason is that they are cosmopolitan and predominant scyphozoan species in many regions worldwide such as northwest Europe [49–51], North America [52], the Black Sea [5], East Asia [3, 46, 53], and Australia [54]. In the same framework, one can also explore other scyphozoan such as *Cyanea nozakii Kishinouye* or the giant jellyfish *Nemopilema nomurai*, the dominant species causing problems in the waters of China [55, 56] and East Asian waters [57].

**Fig 3:**
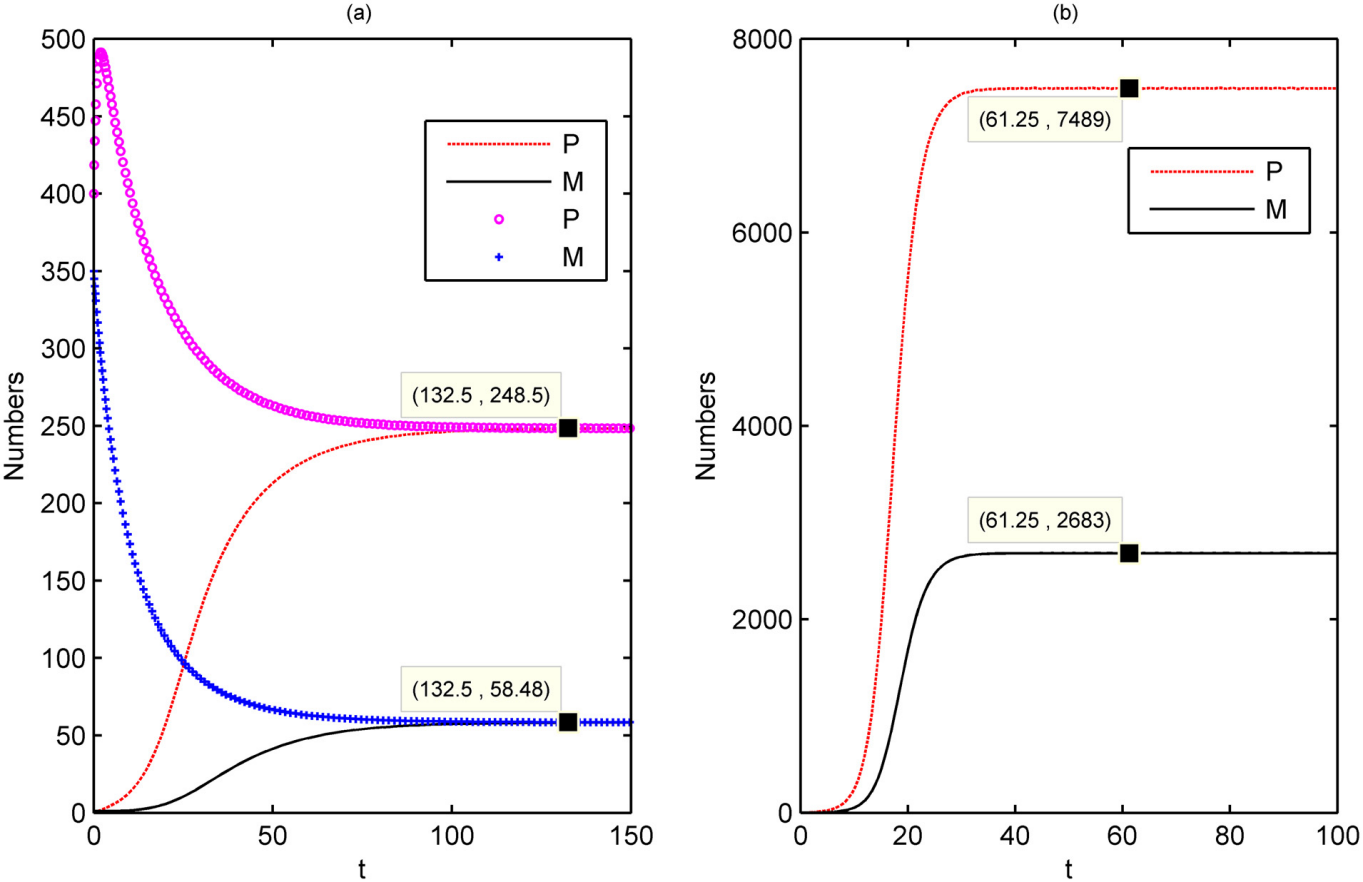
The positive equilibrium E* is globally asymptotically stable. (a) The parameters take the values of V1 in Table 1. (b) The parameters take the values of V2 in Table 1. In this case, the reproduction is high while the losses and competition are low. The solution curves eventually tend to high population level up to 30–50 times of the solution curves in (a) starting from the same initial values (0, 2), which depicts the invaders colonize and multiply from rare species to dominated species.

The positive equilibrium being globally asymptotically stable indicates that, in the long run, the population size will eventually stabilize at a fixed equilibrium, which is independent of the initial values but varies in response to environmental conditions (parameters) (Fig 3). Fig 3(a) and 3(b) depict the cases that the parameters take the values of V1 and V2, respectively. Obviously, the numbers of two stages in Fig 3(a) are lower than those in Fig 3(b). In fact, if the environmental conditions are relatively optimal (e.g., the reproduction being high while the loses and competitions being low in Fig 3(b)), the population sizes abruptly shoot up in a very short time even the initial population sizes are very small. To some extend, Fig 3(b) can help to explain why only a few invaders (even *P* = 0, *M* = 2) can bring about rapid expansion from rare species to dominated species. [22] indicates such a case that *Aurelia* sp.1 is endemic to the western North Pacific and disperses globally from Japan likely related to the historical shipping activity.

Next, we numerically explore the impact of several key limiting factors such as temperature, substrate, and predation on the population dynamics of scyphozoan.

**Fig 4:**
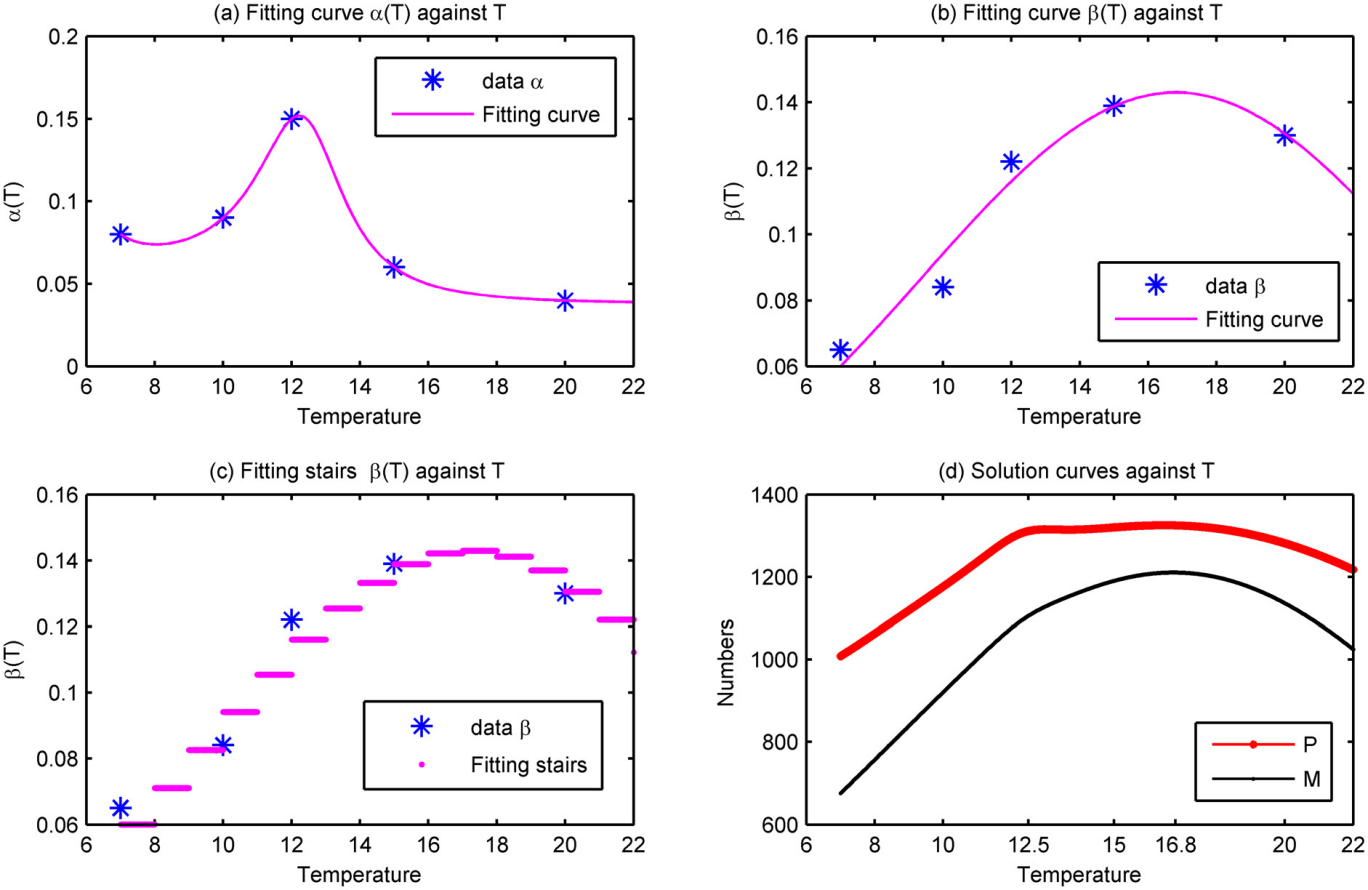
Impact of temperature on steady state population sizes. (a) and (b) are fitting curves of *α*(*T*) and *β*(*T*), where the stars depict the experimental data from [42] standardized by days. (c) is a fitting stairs analogue to (b), which indicates that the temperature warming 1°C leads to the varying of the number of ephyrae. (d) is the bifurcation picture of the population size with respect to temperature, where *b*_1_ = 0.0012, *α*(*T*) and *β*(*T*) are given by Eqs (3) and (4), respectively, and the other parameter values are given by V_2_ in Table 1. The peaks of the population size of polyps and medusae occur at 12.5°C and 16.8°C, respectively. The high temperature is detrimental to the populations and leads to the decrease of population level.

Temperature, one of the important motivating factors that affects asexual reproduction of the scyphozoan, is considered first. We perform a sensitivity analysis as well as a parameter optimization to fit the experimental data. In Fig 4, the budding rate *α*(*T*) and stroibilation rate *β*(*T*) are fitted to the data from [42], where salinity is fixed at 27. After standardized by days (the details are presented in S1 File), the experiment data is plotted in Fig 4 and is marked by stars. Fig 4(a) and 4(b) depict the optimal fitted curves of *α*(*T*) and *β*(*T*) with respect to *T* ∈ [7, 22], where

$$\alpha \left(T\right)=\frac{1.9272}{T^{3}-30.3904T^{2}+294.7234T-871.29}+0.0378,$$ (3)

$$\beta \left(T\right)=0.1430\exp\{-{\left(\frac{T-16.8108}{10.5302}\right)}^{2}\}.$$ (4)

The fitting is pretty good and the summed square of residuals (usually labeled as SSE) are 1.448 × 10^−12^ and 1.625 × 10^−4^, respectively.

Fig 4(c) is an analogue fitting stairs to Fig 4(b), where the stairs indicate that the increase of temperature (per 1°C warming) leads to the variation of ephyrae. The mean value of the relative growth rate from 7°C to 15°C is 12.1%, which is a little bit more than the experimental result 11.3% in [42]. However, from Fig 4(c), the relative growth rates from 7°C to 9°C reach 18.3% and 16.2% for every increased degree of temperature. The relatively high growth rates at low temperature coincide with the phenomena *in situ* observed in [52], the Cornet Bay marina, Washington, USA, where the strobilation occurs from January to April and the temperature varies from 7.6°C to 9°C. So, in some sense, the fitting well depicts the actual case.

Fig 4(d) represents the variation of steady state population sizes with temperature. From Fig 4(d), the number of polyps reaches its peak at *T* = 12.5°C, which corresponds to the maximum budding rate of experimental data. Then the population size of polyps almost remains constant until *T* = 16.8°C, at which the medusae monotonously increases to its maximum level. The difference induced by temperature is reasonable. Because, compared with the strobilation, the budding occurs in lower temperature range and clonal expansion makes it possible to release more ephyrae under high temperature. In addition, Fig 4(d) reveals that continuous high temperature may be detrimental to the population. Although the medusae peak theoretically appears at 16.8°C, which is higher than the experimental result 15°C, our theoretical finding is still significant since the experiment was done only at several typical temperatures instead of continuous temperatures spectrum.

**Fig 5:**
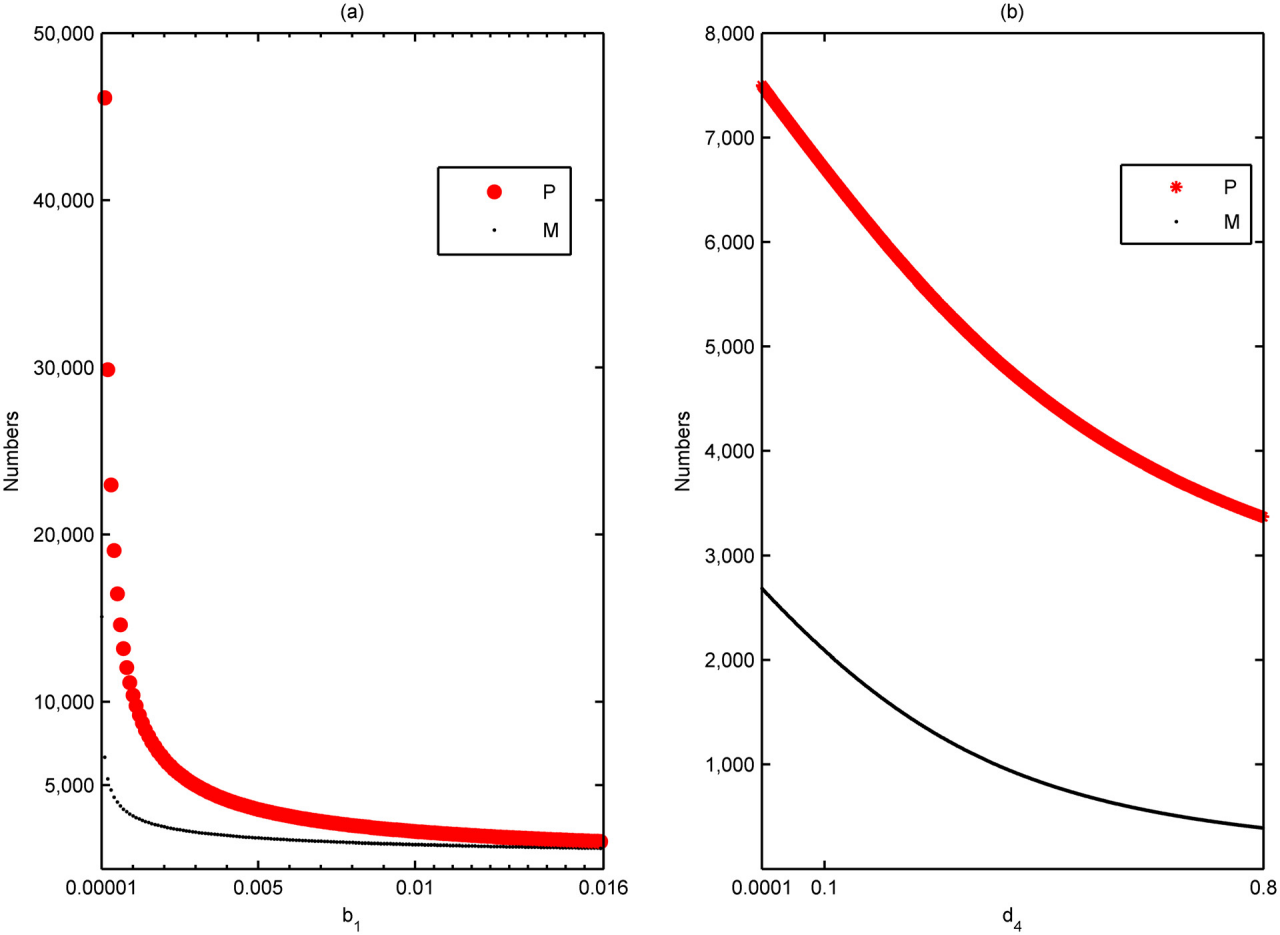
Impact of substrate and predation on steady state population sizes. (a) Steady state population sizes of polyp and medusa with respect to *b*_1_ (the intra-specific competition between polyps). (b) Steady state population sizes of polyp and medusa with respect to *d*_4_ (the mortality rate of medusa induced by inter-specific predation). The values of parameters are given by V_2_ in Table 1 except *s*_1_ = 0.2 in (a).

Fig 5(a) depicts the impact of *b*_1_ on the steady state population sizes or numbers of polyp and medusa. With the decreasing of intraspecific competition between polyps (*b*_1_), the population explodes abruptly. We also note that, as *b*_1_ decreases from 0.1 to 0.0001, the number of polyps and medusae increases from 660 to 49000 and from 660 to 6900, respectively. Fig 5(a) only draws a part of the curves (i.e., *b*_1_ ∈ (0, 0.016]). The whole curves for *b*_1_ ∈ (0, 0.1] shows that, when *b*_1_ > 0.016, the increasing of *b*_1_ has little effect on the variation of population sizes; for 0 < *b*_1_ < 0.016, the change is obvious. The value 0.016 of *b*_1_ is just the reciprocal of the maximum carrying numbers (62 ind/cm^2^) [48].

The numerical simulation further justifies that the intraspecific competition between polyps are mainly the spatial competition (i.e., substrate colony). The decreasing of *b*_1_, which means the intraspecific interactions of polyps for the spatial competition grows weaker (for example, due to the substrate extending), implies that the rate of successful settlement of planulae (*s*_1_) increases. In Fig 5(a), *s*_1_ is set to be 0.2.

Fig 5(b) represents the relations of steady state population sizes of polyp and medusa with respect to *d*_4_. We observe that, with the decreasing of the predation on medusae (*d*_4_), the number of polyps increases simultaneously, even the average increasing speed is higher than that of medusae. Whence, the decrease of predation benefits the growth rate of jellyfish in the pelagic stage.

## Results and Discussion

We have formulated a two-state life history model governed by ODEs is derived to characterize the population dynamics of scyphozoan. Despite its relative simplicity, we offer some hypotheses generated from numerical experiments which may inform future empirical and theoretical experimentation, as well as an extension to the model which may allow for more robust theoretical exploration of jellyfish ecology. Our theoretical analyses and numerical experiments yield several insight into the nature of the model and the system it represents. We found that the polyp-medusa model admits trichotomous global dynamic scenarios: either *E*_0_, or *E*_1_, or E* being globally asymptotically stable. The analytical and numerical analyses elucidate that the population dynamics sensitively depend on several key limiting factors such as substrate, temperature, and predation.

Artificial structures provide new habitats for planula settlement, extend the distribution of scyphozoan species, give rise to the decreasing of *b*_1_ and the increasing of *s*_1_, and result in the increasing frequency of jellyfish blooms. Many studies focus on the important role of substrates playing in the population dynamics of jellyfish, among which, the majority focus on the preference of polyps for different substrate types, natural or artificial [12, 48, 58], and others illustrate that the possible link between ocean sprawl and jellyfish proliferation is crucial for expounding the mechanism of jellyfish blooms [20, 21, 54]. Our theoretical study confirms that the scyphozoan species are explicitly and sensitively affected by the expansion of substrates and the benthic phase of scyphozoan life cycle contributes much more to the abundance of the medusa. The potential that artificial structures serving as substrate for polyps in jellyfish blooms must be considered in practical management.

Temperature is one of the most important triggering factors of jellyfish blooms. This study reveals that, within a suitable range of temperature, the increasing of temperature might lead to the explosion of polyps and medusae, but much higher temperatures would be detrimental to both forms. This finding is consistent with the experiment results in [46]. It is well known that the global warming has caused the ocean water to warm rather erratically and has proved to have negative effects for many species regulating jellyfish such as Leatherback and specialized fish species while has helped jellyfish thrive [1, 17]. This accounts for possible the fact that ongoing increases in water temperature result in the appearance of adult jellyfish swarms.

In addition to the problems due to temperature change, the decline of predation may also accelerate the rapid growth of jellyfish. Many predators regulating jellyfish numbers are victims of human activities such as overfishing [59]. Some adult fish are among the main predators of jellyfish, their rapid disappearance has allowed the jellyfish population to skyrocket [1]. In addition, the unrestricted fishing has reduced the number of creatures that jellyfish compete with for plankton and other forms of food and is resulting in a decreased level of competition for the jellyfish, which also fuels the growth of the jellyfish population [11]. Increasing and enforcing fishing regulations must be on the way. One more method to control the jellyfish blooms is to increase the predation by introducing other gelatinous zooplankton [24, 60] or jellyfish-eating fish such as silver pomfret *Pampus argenteus* [61].

In summary, jellyfish blooms seem to be related to human induced stresses like predation fading, habitat modification, and climate (temperature) change. The combination of these factors acts synergistically to create a habitat that is more favorable for jellyfishes. Reducing artificial marine constructions, aiding predator populations, and directly controlling the jellyfish population would all, if implemented with an adaptive management style plan, help to manage the jellyfish blooms. This theoretical study provides insight into the facts underlying this model, illuminates the triggering mechanisms of jellyfish blooms, and sheds some new light on the general control strategy for jellyfish.

Although the numerically simulations are carried out for a specified species *Aurelia* sp., the idea and approach fall into a general framework that can be applied to other scyphozoan species and compensate the experimental studies. The reason is that both empirical and field experiments are case-related and limited by experimental circumstance. For example, *A. aurita* lives in much warmer temperatures (19 ∼ 32°C) in Taiwan [46], a relatively colder range (7 ∼ 15°C) in the northeast Pacific, and in a wider temperature range (5 ∼ 30°C) in Japan [28]. Moreover, the experiments are usually done only for several typical temperature scenarios instead of continuous temperature spectrum.

In this study, predation is simply included implicitly as constant per capita death rate induced by predators, rather than being subsumed as a dependent trophic variable input to scyphozoa populations. While this is a very strong assumption, it allows us to focus solely on the scyphozoan life cycle and several typical limiting factors. More biotic and abiotic factors and processes must be incorporated into the dynamic modeling of scyphozoan and marine ecosystems. It will be more interesting but more challenging.

## Supporting Information

S1 File Estimation of model parameters. We deal with the dimensional homogeneity by standardizing the data by days since different dimensions are adopted in different experiments. The estimated parameter values or ranges are list in Table 1.
(PDF)

S2 File Existence and stability of equilibria of system (2). The standard qualitative analysis techniques help to prove the existence and stability of the equilibria and the global dynamics of system (2).
(PDF)
