# Supplementary material for: Dynamic Model for Life History of Scyphozoa
Source: PLoS One. 2015 Jun 26;10(6):e0130669. doi: 10.1371/journal.pone.0130669 (PMC4482707; doi:10.1371/journal.pone.0130669)
Supplement: S1 File — We deal with the dimensional homogeneity by standardizing the data by days since different dimensions are adopted in different experiments. The estimated parameter values or ranges are list in Table 1. (PDF) [file pone.0130669.s001.pdf]

Supporting Information

S1 File. Estimation of model parameters.

Here we deal with the dimensional homogeneity by standardizing the data by days since different dimensions are adopted in different experiments. The estimated parameter values or ranges are list in Table 1.

For *Aurelia* sp., the budding is the mainly asexual reproduction of polyp (94% of the total), while stolon and podocyst account for only 5 and 1%, respectively [1]. Moreover, podocysts are usually produced when the food supply is at low level, which contradicts with the assumption that the resource is sufficient. Hence, when the discussions focus on *Aurelia* sp., the asexual reproduction rate $\alpha(T)$ represents the budding rate. However, when one deals with *Cyanea nozakii Kishinouye* or *Nemopilema nomurai*, $\alpha(T)$ emphasizes the podocyst rate.

The budding rate $\alpha(T)$ and strobilation rate $\beta(T)$ are derived from [2]. The experiment was operated with *Aurelia labiata* in Puget Sound, Washington, USA, to test the responses at the extremes of local environmental conditions with $10-12^{o}$C temperature and 27 salinity (typical conditions beneath the surface layer). The polyps were fed in excess twice weekly. The data provided are about of the mean number of buds or strobilations produced by per polyp in different temperature. Note that polyp is perennial and the asexual reproduction can take place from time to time, so the data are divided by experimental period and $\alpha(T)$ is obtained. For the strobilation rate $\beta(T)$, the reproduction period is completely included in the experimental period, so the data are divided by 365 days and other strobilation period in the same year is adjusted by the times n. It's worth mentioning that, if the survival period of polyp is less than one year, then to standardize the data is just to divide the number of buds or ephyrae by the survival period, just like that done in [3].

For γ, [4, 5] indicate that a mature medusa releases $7000 \sim 65000$ planulae every time . After standardizing the data, one obtains $\gamma \in (19, 178)$. In [5], *A. aurita* is considered to be an r-strategist and then mortality of planula in the water column is high, which makes the survival rate s_1 being low. However, if the environment is favorable for planula to swim and settle, then s_1 will be increasing to 0.3 [6]. The death rate of ephyra is generally considered to be low [7] and the survive rate s_2 is high if they are lack of natural predators such as *Cyanea nozakii* [8]. So s_1 and s_2 can be estimated (Table 1).

For d_1, the lifetime of polyp is very long, even up to decades, so d_1 is very small and is even negligible in some cases. However, [3] presented that the survival period of polyp was less than one year in Taiwan, which implied that high temperature was detrimental to polyps. By [3], d_1 lies in $0 \sim 0.028$. For d_2, a dramatic decline in polyp population results from being covered by silt or consumed by the nudibranch [5, 9]. So it is assumed that $d_2 \in (0, 0.3)$. In most environments, medusae live for $4 \sim 8$ months and then deteriorate or die after spawning [5]. From [10], the natural mortality of medusae can be 6.61 even 10.3 year^{-1}, so d_3 can be obtained. For d_4,

although the predators such as turtles and specialized fish species are decreasing, other gelatinous zooplanktons become more important if they meet. In [8], ephyrae and young *Cyanea nozakii* were fed with ephyrae of *Rhopilema esculentum* and *Aurelia aurita* Linneus. In fact, 'jellyfish' has the largest proportion of the main predators of gelatinous organisms (32%) [11]. So d_4 is estimated from [11] as listed in Table 1.

[12] observed that the polyps of *A. aurita* preyed on conspecifics when food availability is low, which contradicts with the assumption that the resource is sufficient. So the intraspecific competition of polyps (b_1) mainly focuses on the spatial resources, which affects the settlement of planulae to the seabed. If the space is limited, b_1 is inversely proportional to the maximum carrying numbers in the unit area, for example 62 per cm^2 [12]. When the substrates extend greatly, b_1 would be very small. So we assume $b_1 \in (0,\ 0.1)$. For b_2, the intraspecific competition even cannibalism has been observed within some scyphozoan in the laboratory such as *Pelagia noctiluca* [13], but this does not be detected in *Aurelia* sp. So, when we consider *Aurelia* sp., b_2 can be ignored.

References

1. Han CH, Uye Si. Combined effects of food supply and temperature on asexual reproduction and somatic growth of polyps of the common jellyfish *Aurelia aurita* sl. Plankton and Benthos Research. 2010;5(3):98–105. Available from: `http://dx.doi.org/10.3800/pbr.5.98`.

2. Purcell JE. Environmental effects on asexual reproduction rates of the scyphozoan *Aurelia labiata*. Marine Ecology Progress Series. 2007;348:183–196. Available from: `doi:10.3354/meps07056`.

3. Liu WC, Lo WT, Purcell JE, Chang HH. Effects of temperature and light intensity on asexual reproduction of the scyphozoan, *Aurelia aurita* (L.) in Taiwan. Hydrobiologia. 2009;616(1):247–258. Available from: `doi:10.1007/s10750-008-9597-4`.

4. Lucas C. Population dynamics of *Aurelia aurita* (Scyphozoa) from an isolated brackish lake, with particular reference to sexual reproduction. Journal of Plankton Research. 1996;18(6):987–1007. Available from: `doi:10.1093/plankt/18.6.987`.

5. Lucas CH. Reproduction and life history strategies of the common jellyfish, *Aurelia aurita*, in relation to its ambient environment. Hydrobiologia. 2001;451(1-3):229–246. Available from: `doi:10.1023/A:1011836326717`.

6. Conley K, Uye Si. Effects of hyposalinity on survival and settlement of moon jellyfish (*Aurelia aurita*) planulae. Journal of Experimental Marine Biology and Ecology. 2015;462:14–19. Available from: `doi:10.1016/j.jembe.2014.10.018`.

7. Schneider G. Estimation of food demands of *Aurelia aurita* medusae populations in the Kiel Bight/western Baltic. Ophelia. 1989;31(1):17–27. Available from: `doi:10.1080/00785326.1989.10430848`.

8. Dong J, Sun M, Wang B, Liu H. Comparison of life cycles and morphology of Cyanea nozakii and other scyphozoans. Plankton Benthos Res. 2008;3(Suppl):118–124. Available from: http://dx.doi.org/10.3800/pbr.3.118.

9. Thiel H. Untersuchungen über die Strobilisation von *Aurelia aurita* Lam. an einer Population der Kieler Förde. Kieler meeresforschungen. 1962;18:198–230.

10. Palomares M, Pauly D. The growth of jellyfishes. In: Jellyfish Blooms: Causes, Consequences, and Recent Advances. Springer; 2009. p. 11–21. Available from: doi:10.1007/978-1-4020-9749-2_2.

11. Pauly D, Graham W, Libralato S, Morissette L, Palomares MD. Jellyfish in ecosystems, online databases, and ecosystem models. Hydrobiologia. 2009;616(1):67–85. Available from: doi:10.1007/s10750-008-9583-x.

12. Gröndahl F. Evidence of gregarious settlement of planula larvae of the scyphozoan *Aurelia aurita*: An experimental study. Marine ecology progress series. 1989;56(1):119–125.

13. Lilley MK, Elineau A, Ferraris M, Thiéry A, Stemmann L, Gorsky G, et al. Individual shrinking to enhance population survival: quantifying the reproductive and metabolic expenditures of a starving jellyfish, *Pelagia noctiluca*. Journal of Plankton Research. 2014;p. 1–13. Available from: doi:10.1093/plankt/fbu079.
