# Supplementary material for: Dynamic Model for Life History of Scyphozoa
Source: PLoS One. 2015 Jun 26;10(6):e0130669. doi: 10.1371/journal.pone.0130669 (PMC4482707; doi:10.1371/journal.pone.0130669)
Supplement: S2 File — The standard qualitative analysis techniques help to prove the existence and stability of the equilibria and the global dynamics of system (2). (PDF) [file pone.0130669.s002.pdf]

# Supporting Information

## S2 File. Existence and stability of equilibria of system (2)

**Theorem 1.** $\Omega := \{(P,M) \in \mathbb{R}_+^2 : 0 < P < l, 0 < M < cl/d\}$ *is positively invariant with respect to system (2).*

*Proof.* We prove it by phase plane analysis. Consider the vector field defined on the boundary of $\Omega$. On the bottom boundary of $\Omega$ ( i.e., $\{(P,M) \in \mathbb{R}^2 : 0 < P < l, M = 0\}$), one has $\dot{M} = cP \geq 0$. On the upper boundary of $\Omega$ (i.e., $\{(P,M) \in \mathbb{R}^2 : 0 < P < l, M = cl/d\}$), $\dot{M} < -b_2 M^2 < 0$. On the left boundary of $\Omega$ ( i.e., $\{(P,M) \in \mathbb{R}^2 : P = 0, 0 < M < cl/d\}$), $\dot{P} = bM > 0$. On the right boundary of $\Omega$ ( i.e., $\{(P,M) \in \mathbb{R}^2 : P = l, 0 < M < cl/d\}$), $\dot{P} < (a - |a|)P \leq 0$. Therefore, any trajectory starting in $\Omega$ stays in $\Omega$ for all forward time. ☐

**Theorem 2.** *System (2) has no closed orbit in* $\Omega$ *.*

*Proof.* Consider the Dulac's function $H(P,M) = 1/P$, then

$$\frac{\partial(F_1 H)}{\partial P} + \frac{\partial(F_2 H)}{\partial M} = -b_1 - \frac{bM}{P^2} - \frac{d}{P} - \frac{2b_2 M}{P} < 0$$

By Bendixson-Dulac's negative criterion [1], there cannot be a closed orbit contained in $\Omega$. ☐

**Theorem 3.** $E_0(0,0)$ *always exist,* $E_1(a/b_1, 0)$ *exists if and only if* $ad + bc > 0$ *and* $c = 0$ *(i.e.* $a > 0$ *and* $c = 0$*), and the positive equilibrium* $E^*(P^*, M^*)$ *exists if and only if* $ad + bc > 0$ *and* $c \neq 0$.

*Proof.* The equilibrium $(P,M)$ of (2) solves

$$aP + bM - b_1 P^2 = 0, \quad cP - dM - b_2 M^2 = 0. \tag{$\bigstar$}$$

$E_0$ obviously exists. If $a > 0$ and $c = 0$, then solving ($\bigstar$) produces $E_1$; Otherwise, there is no $E_1$ solving ($\bigstar$).

Next, we deal with the existence of the positive equilibrium $E^*$.

If $c = 0$, then it is obvious that $E^*$ does not exist.

If $ad + bc \leq 0$, then we have $a < 0$. From ($\bigstar$), it follows that

$$F(P) = b_1^2 b_2 P^3 - 2ab_1 b_2 P^2 + (a^2 b_2 + bb_1 d)P - b(ad + bc) = 0.$$

Then

$$F'(P) = 3b_1^2 b_2 P^2 - 4ab_1 b_2 P + (a^2 b_2 + bb_1 d) > 0 \quad \text{for } P > 0.$$

Note that $F(0) = -(ad + bc) \geq 0$, then $F(P) > 0$ and $F(P) = 0$ if and only if $P = 0$. Therefore, if $ad + bc \leq 0$, then $E^*$ does not exist.

If $ad+bc>0$, $c\neq 0$, and $b=0$, then solving ($\bigstar$) gives a unique $E^*$ with

$$P^*=\frac{a}{b_1}>0,\quad M^*=\frac{-b_1d+\sqrt{b_1^2d^2+4acb_1b_2}}{2b_1b_2}>0.$$

If $ad+bc>0$, $c\neq 0$, and $b\neq 0$, then $F(0)=-(ad+bc)<0$. If $a\leq 0$, then $F'(P)>0$ and

$$F(\frac{bc}{b_1d})=\frac{b^3b_2c^3}{b_1d^3}-\frac{2ab^2b_2c^2}{b_1d^2}+\frac{a^2b_2bc}{b_1d}-abd>0.$$

By the continuity and monotonicity of $F(P)$, there exists a unique $P^*\in(0,bc/b_1d)$ such that $F(P^*)=0$ and then $M^*=(b_1P^{*2}-aP^*)/b>0$. If $a>0$, then

$$F(a/b_1)=-b^2c<0,\quad F(\frac{ad+bc}{b_1d})=\frac{b_2}{b_1d}(ad+bc)(\frac{bc}{d})^2>0$$

and

$$F'(a/b_1)=bb_1d>0,\quad F'(P)=3b_1^2b_2(P-2a/3b_1)^2+1/3(3bb_1d-a^2b_2)$$

is increasing in $(a/b_1,(ad+bc)/b_1d)\subset(2a/3b_1,+\infty)$. Whence, $F'(P)>0$ for $P\in(a/b_1,(ad+bc)/b_1d)$. By the continuity and monotonicity of $F(P)$, there exists a unique $P^*\in(a/b_1,(ad+bc)/b_1d)$ such that $F(P^*)=0$ and then $M^*=b_1P^*(P^*-a/b_1)/b>0$. The proof is complete. □

The Jacobian of system (2) evaluated at $(P,M)$ reads

$$J(E)=\begin{pmatrix} a-2b_1P & b \\ c & -d-2b_2M \end{pmatrix}.$$

**Theorem 4.** *If $ad+bc<0$, then $E_0$ is globally asymptotically stable node; if $ad+bc=0$, then $E_0$ is a saddle-node and the saddle-node bifurcation occurs; if $ad+bc>0$, then $E_0$ is an unstable saddle.*

*Proof.* Note that

$$\mathrm{Tr}(J(E_0))=a-d,\quad \det(J(E_0))=-(ad+bc).$$

If $ad+bc<0$, then $\mathrm{Tr}(J(E_0))<0, \det(J(E_0))>0$. Hence $E_0$ is locally asymptotically stable. In addition, in this case, $E_0$ is the unique equilibrium. By Theorem 2 and Poincare-Bendixson Theorem, $E_0$ is globally asymptotically stable. The proof of the rest of the claims is trivial. The details are omitted. □

**Theorem 5.** *If $ad+bc>0$ and $c=0$ (i.e. $a>0$ and $c=0$), then $E_1$ is globally asymptotically stable.*

*Proof.* Note that $\mathrm{Tr}(J(E_1))=-(a+d)<0$, $\det(J(E_0))=ad>0$. Then, $E_1$ is locally asymptotically stable. In this case, $E_0$ is unstable saddle and $E^*$ does not exist. The conclusion follows from Theorem 2 and Poincare-Bendixson Theorem. □

**Theorem 6.** *If $ad+bc>0$ and $c\neq 0$, then $E^*$ is globally asymptotically stable.*

*Proof.* It is not difficult to show that

$$J(E^*) = \begin{pmatrix} -bM^*/P^* - b_1P^* & b \\ c & -cP^*/M^* - b_2M^* \end{pmatrix},$$

then $\mathrm{Tr}(J(E^*)) < 0$ and $\det(J(E^*)) > 0$. Hence, $E^*$ is a node and is locally asymptotically stable. In this case, $E_0$ is an unstable saddle and $E_1$ does not exist. Therefore, since $\Omega$ is simply connected and positively invariant and contains no closed orbits, by Poincaré-Bendixson Theorem [1], all solutions of (2) starting in $\Omega$ converge to $E^*$. Thus, $E^*$ is globally asymptotically stable. □

# References


1. Zhang Z, Ding T, Huang W, Dong Z. Qualitative Theory of Differential Equations. 1992. Amer Math Soc, Providence;.
